# Mapping the basal ganglia alterations in children chronically exposed to manganese

**DOI:** 10.1038/srep41804

**Published:** 2017-02-03

**Authors:** Yi Lao, Laurie-Anne Dion, Guillaume Gilbert, Maryse F. Bouchard, Gabriel Rocha, Yalin Wang, Natasha Leporé, Dave Saint-Amour

**Affiliations:** 1 CIBORG laboratory, Department of Radiology, Children’s Hospital Los Angeles, Los Angeles CA, USA; 2 Department of Biomedical Engineering, University of Southern California, Los Angeles CA, USA; 3 Department of Psychology, Université du Québec à Montréal, Montréal, QC, Canada; 4 Department of radiology, Université de Montréal, Montréal, QC, Canada; 6 MR Clinical Science, Philips Healthcare, Montreal, Quebec, Canada; 5 Sainte-Justine Hospital Research Centre and Department of Occupational and Environmental Health, Université de Montréal, Montréal, QC, Canada; 7 School of Computing, Informatics, Decision Systems and Engineering, Arizona State University, Tempe, Arizona, USA

## Abstract

Chronic manganese (Mn) exposure is associated with neuromotor and neurocognitive deficits, but the exact mechanism of Mn neurotoxicity is still unclear. With the advent of magnetic resonance imaging (MRI), *in-vivo* analysis of brain structures has become possible. Among different sub-cortical structures, the basal ganglia (BG) has been investigated as a putative anatomical biomarker in MR-based studies of Mn toxicity. However, previous investigations have yielded inconsistent results in terms of regional MR signal intensity changes. These discrepancies may be due to the subtlety of brain alterations caused by Mn toxicity, coupled to analysis techniques that lack the requisite detection power. Here, based on brain MRI, we apply a 3D surface-based morphometry method on 3 bilateral basal ganglia structures in school-age children chronically exposed to Mn through drinking water to investigate the effect of Mn exposure on brain anatomy. Our method successfully pinpointed significant enlargement of many areas of the basal ganglia structures, preferentially affecting the putamen. Moreover, these areas showed significant correlations with fine motor performance, indicating a possible link between altered basal ganglia neurodevelopment and declined motor performance in high Mn exposed children.

Manganese (Mn) is an essential element that participates in daily metabolic activities in the human body, but that can be toxic when the dose exceeds Mn homeostasis^1^,^2^. Studies have shown that Mn accumulation due to chronic occupational or environmental exposure may cause neuromotor and cognitive deficits^3^,^4^,^5^,^6^. There are growing concerns about the risk of Mn childhood accumulation^7^,^8^,^9^,^10^,^11^,^12^,^13^, as the immaturity of the biliary system results in higher Mn retention in both young animals and humans^14^,^15^. In particular, Wasserman *et al*. investigated 10-year old children and found that children consuming drinking water with a high concentration of Mn had significantly lower IQ scores^7^. Bouchard *et al*.^11^ further confirmed the adverse effects of Mn accumulation via drinking water on cognition, and these effects were observed in lower water Mn concentrations than in Wasserman *et al*.^7^. With the advent of MRI, efforts have been made to elucidate the neuro-substrates for Mn neurotoxicity. A brain diffusion tensor imaging (DTI) study of adult welders showed significant alterations of the corpus callosum (CC) and frontal white matter from long-term Mn exposure^16^. Task fMRI studies indicated additional brain activations in adult welders compared with controls, likely to ensure adequate motor performance and working memory^17^,^18^. However, the exact mechanisms of Mn neurotoxicity are still not fully understood, especially in cases of exposure to Mn through drinking water, and the determination of dose-response level of Mn toxicity remains elusive^1^. More dedicated studies on selective brain regions are needed to develop biomarkers of excessive Mn exposure and their association with clinical symptoms.

The basal ganglia (BG) consists of several important subcortical gray matter nuclei, and is thought to be the intersection where action and cognition meet^19^. Due to its involvement in several motor and cognitive cortical-subcortical loops, BG dysfunctions often lead to disorders of motor initiation and inhibition, notably Parkinson’s disease (PD)^20^,^21^. Mn intoxication patients often show symptoms that resemble those of PD, therefore the BG has been targeted as a putative location for studies on manganese toxicity^2^,^22^. Among the several sub-nuclei of the BG, the globus pallidus is thought to be the most affected, but inconsistent results have been reported. For instance, Hauster *et al*.^23^, Fell *et al*.^24^, Kafritsa *et al*.^25^, and Ikeda *et al*.^26^ found T1 hyperintensities in the globus pallidus in patients with elevated Mn blood concentrations due to chronic liver disease or long-term parenteral nutrition, while Shinotoh *et al*.^27^, Kim *et al*.^28^ as well as Aschner *et al*.^29^ reported no BG T1 signal intensity alterations in patients with occupational Mn exposure or manganese intoxication secondary to iron deficiency anemia. In addition, Criswell *et al*. explored several BG subregions and found that intensity indices in the caudate, the anterior and posterior putamen and the globus pallidus were all correlated with the magnitude of Mn exposure^30^. More interestingly, the combined global basal ganglia intensity index was more correlated with Mn exposure than the widely accepted pallidal index. The discrepancy in previous studies may be due to varied Mn toxicity levels, and methodologies that are either not powerful enough or whose results vary with MRI scanner settings. Thus, higher detection power techniques are required to better understand the relative vulnerability of the BG nuclei to Mn toxicity.

Here, for the first time, we apply a 3D surface-based morphometry analysis on 3 BG structures, including the putamen, the globus pallidus and the caudate, to investigate the neuroanatomic correlates of chronic childhood Mn exposure through drinking water. In the present study, we compare 10 high Mn exposure and 13 age matched low exposure school-age children, and aim to examine the differences associated with Mn exposure through drinking water. We previously assessed these children with the standard MRI procedure for Mn exposure, that is, evaluation of MRI signal intensity in the globus pallidus. We did not observe any hyperintensities in the globus pallidus, as might be expected based on the literature on occupationally exposed individuals^31^, but rather, as detailed in our previous work, we observed significant hypointensities in the group of children with higher Mn exposure^13^. The significance of this finding is not clear, but might indicate neurological damages since the hypointensities correlated with poorer motor function^13^. Our study may provide new biomarkers for Mn neurotoxicity as well as neuro-substrates for the impaired motor performance associated with excessive Mn exposure.

## Results

**Figure 1:**
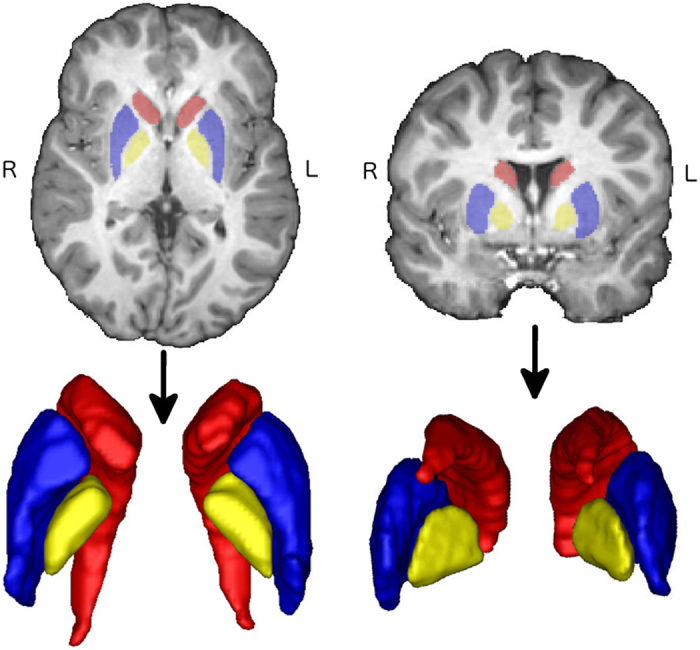
Illustration of segmentation and the corresponding 3D structures in axial and coronal views. Red, blue and yellow each represents the caudate, the putamen and the globus pallidus, respectively. None of the participants had visible hyper- or hypointensities in the basal ganglia.

Twenty-three children aged 9 to 15 (mean: 12.2 years) were grouped into 10 high Mn exposure and 13 age matched low exposure participants, according to the Mn concentration in their drinking water. Brain T1 MR images of all the subjects were acquired using a 3T Philips scanner. Three bilateral BG nuclei (the caudate, the putamen, and the globus pallidus) were manually traced on preprocessed and linearly aligned T1 images. In the presented dataset, no visible T1 intensity alterations were found in the BG in our participants. Thus, potential Mn depositions in the deep gray matter structures, if there were any, did not influence the differentiation of BG nuclei boundaries. Based on the binary segmentations, 3D surface mesh grids of the BG structures were constructed and registered using an in-house algorithm^32^. Manual segmentation of 3 bilateral BG nuclei and the corresponding 3D surfaces are shown in Fig. 1. This was followed by a traditional volume based analysis (VBA) and a multivariate tensor based morphometry (mTBM). Details can be found in the materials and methods section.

Group analysis results using VBA and mTBM are shown in Table 1. Whole structure volumes are computed to provide an intuitive comparison of structure sizes between the low and high Mn exposure groups, while mTBM is more sensitive to regional alterations in structures. As we can see from Table 1, all the investigated BG structures (the putamen, the globus pallidus and the caudate) have average volumes that are slightly larger in the high Mn exposure group compared with those from the low Mn exposure group. However, none of these differences reach statistical significance. As for surface based morphometry, after structure-wise multiple comparisons correction, the bilateral putamen shows significant differences between exposure groups, and the bilateral caudate and globus pallidus both show similar trends. We also run structure-wise permutation based statistical tests on both sides of the structures to correct for multiple comparisons in an exploratory analysis. We detect significant differences in the left putamen and trends in the right putamen as well as the left globus pallidus.

**Figure 2:**
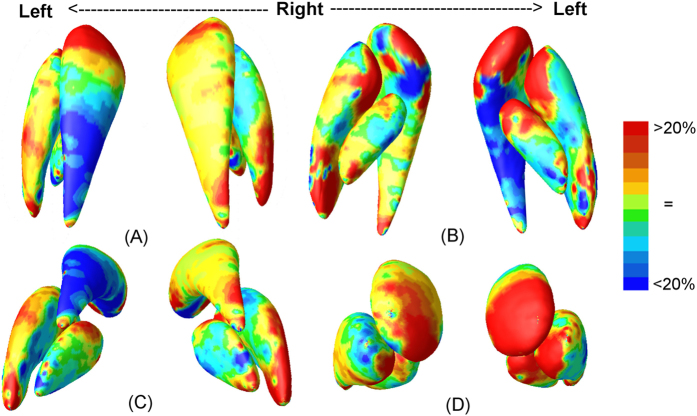
Mean ratio map of det *J* between high-exposed and low-exposed groups are displayed in four views: superior (**A**), inferior (**B**), posterior (**C**), and anterior (**D**). Areas in red represent an increase of det *J* in the high-exposed group compared with that in the low-exposed group, indicating an expansion of the corresponding areas. Areas in blue show a decrease *detJ* in the high-exposed group compared with that in the low-exposed group, indicating a contraction of the corresponding areas.

**Figure 3:**
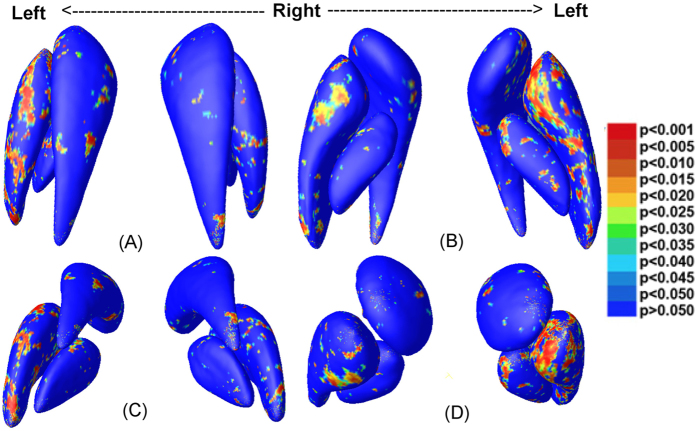
Statistical results of the multivariate analysis are displayed in four views: superior (**A**), inferior (**B**), posterior (**C**), and anterior (**D**). Areas in colors other than deep blue represent vertex-wise significances of the multivariate analyses (p < 0.05).

To visualize the type of alterations (i.e., enlargement or shrinkage) in surface based morphometry, we map the ratio of the mean determinants (det *J*) of the two groups at each vertex (Fig. 2). Vertex-wise multivariate analyses based on the combination of 4 feature values (MAD and 3 eigenvalues of the logged deformation tensor) are also performed, and the corresponding p-value maps are shown in Fig. 3. While for several of the BG subnuclei (i.e., the right putamen and the left globus pallidus), structure-wise p-values in Table 1 only show trends, significant clusters with evident enlargements are present in several sub-regions on the nuclei’s surfaces.

As shown in Figs 2 and 3, several areas of the BG structures, notably the anterior aspect, are significantly enlarged. In the putamen, both the anterior and the posterior ends are larger in the high-exposure group, and broad surface areas show significant group differences in Fig. 3. In the globus pallidus, most of the significant clusters are larger in the high-exposure group, with the main significant clusters located in the left anterior side. In the caudate, we observe evidently larger anterior ends on the bilateral sides, and a smaller posterior end of the left side in the high-exposure group, though only few surface clusters reach significance.

**Figure 4:**
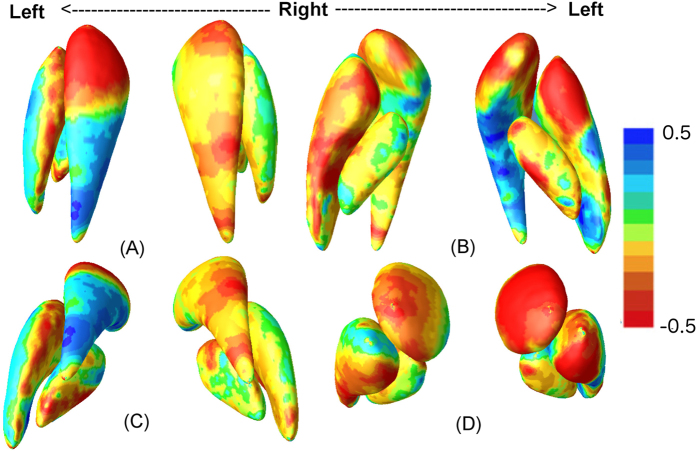
Vertex-wise correlation coefficients between vertex-wise det *J* values with Santa Ana scores are displayed in four views: superior (**A**), inferior (**B**), posterior (**C**), and anterior (**D**). Areas in red colors represent a negative correlation between surface areas and motor performance, and vice versa for blue colors.

**Figure 5:**
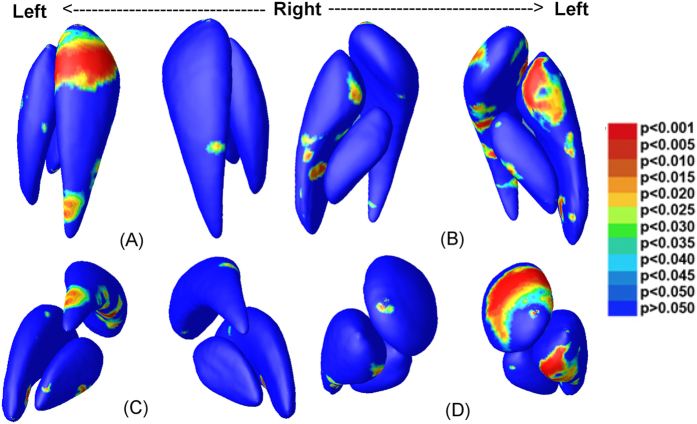
Correlation of vertex-wise det *J* values with Santa Ana scores are displayed in four views: superior (**A**), inferior (**B**), posterior (**C**), and anterior (**D**). Areas in colors other than deep blue represent vertex-wise significances of the multivariate analyses (p < 0.05).

Vertex-wise *detJ* from mTBM are also correlated with motor performance, specifically the Santa Ana Pegboard Test developed to assess fine motor function^33^. In Figs 4 and 5, structural enlargement, notably the anterior aspect of the left putamen and the left caudate are negatively correlated with motor performance. Moreover, areas significantly correlated with motor performance largely overlap those shown in the group difference maps in Figs 2 and 3.

## Discussion

Here, we applied a MRI based 3D mTBM analysis on BG subnuclei to investigate BG morphometry alterations in the developing brain in response to long-term Mn exposure from drinking water. Statistical comparisons based on BG subnuclei volume did not detect any significant alterations. Using mTBM, we successfully detected significant enlargement in the putamen and trends of enlargement in the left globus pallidus. In addition, we correlated fine motor performance, based on Santa Ana test scores, with regional surface measurements of det *J*. Reduced motor performance was found to be significantly correlated with regional enlargement in anterior ends of the putamen, globus pallidus and caudate – the same areas that were also found to be significantly altered in the mTBM comparisons between Mn exposure groups. These results are in accordance with deficits in cognitive and neuromotor performance observed in these children from previous studies^12^,^13^, and with a study showing that enlarged anterior BGs are associated with lower behavioral performance in pre-adolescent children^34^.

Our findings demonstrated significant regional enlargement in BG subnuclei of Mn exposed children. Altered BG morphologies are significantly associated with poor motor performance in this population. Collectively, these results suggest that neuro-circuits within the motor loop are at risk in children with Mn exposure through drinking water.

Although there is still controversy regarding the exact mechanisms of Mn toxicity, growing focus has been directed to dysfunctions of the basal ganglia motor pathways^27^,^35^,^36^. The striatum, including the caudate and the putamen, receive anatomical projections from the motor cortex and substantia nigra pars compacta and send outputs via the globus pallidus, forming programming motor loops. The striatum and the globus pallidus are thought to be the primary targets of Mn accumulation in the brain^2^. In particular, a positron emission tomography (PET) study on four patients with chronic occupational Mn exposure proposed that Mn is likely to disrupt downstream BG dopaminergic output pathways^27^. Criswell *et al*. investigated 20 asymptomatic welders exposed to Mn fumes, and reported reduced 6-[18F]fluoro-L-DOPA (FDOPA) PET uptake predominantly in the anterior striatum^35^. A subsequent study on a patient with Mn accumulation in end-stage liver disease confirmed reduced FDOPA uptake in the caudate, and in the anterior and posterior putamen^36^. In addition, a voxel based morphometry study reported volume reduction co-located to the globus pallidus^37^, while other T1 intensity based studies on the globus pallidus reported inconsistent findings^23^,^24^,^25^,^26^,^27^,^28^. Our current study extends these findings on the Mn-induced neuroanatomical changes in the BG subnuclei to children with exposure to Mn present in drinking water. Here, we detected evident enlargements in BG subnuclei, notably the left caudate, the whole putamen and the globus pallidus. Concurrent alterations in all three regions may imply disturbances in their shared BG motor pathways.

Support for the hypothesis of BG motor pathways disturbances also comes from our correlation analysis between BG shape measurements and fine motor performance. In a previous study in the same population^13^, three different motor measurements were tested, and Santa Ana Pegboard Test performances were the ones most correlated with Mn exposure. In line with this, in the presented study, enlargements in the anterior caudate and putamen were significantly correlated with Santa Ana scores. These are also consistent with the findings of Sandman *et al*.^34^, in which anterior striatum changes were found to be significantly associated with poorer cognition in healthy pre-adolescent children. However, the current study is not powered to reveal disturbances in specific motor pathways (i.e. direct/indirect motor pathways, or the nigrostiatal pathways.). Future investigations integrating high angular resolution diffusion - weighted imaging (HARDI) tractography analysis on selected region of interest will be needed to refine our current findings.

While previous MRI studies of Mn neurotoxicity focused on occupational Mn exposure in adults, we are the first to investigate basal ganglia morphometry alterations in children with Mn exposure. Studies of Mn intoxication in adults have reported reduced gray matter volumes as well as reduced WM integrity^37^. For instance, in a whole brain voxel-based morphometry analysis^37^, brain volumes, notably the globus pallidus and cerebellar regions, were found to be reduced in occupationally Mn exposed welders. A region of interest study that was also performed on welders showed reduced apparent diffusion coefficient values in the globus pallidus and anterior putamen^30^. The enlargement of bilateral BG structures in our study are opposite in direction to the reductions found in adults. The discrepancy may be due to the different age of the included subjects, coupled with different Mn exposure routes^37^.

The brain undergoes important development from childhood to adolescence, and involves a complex sequence of neuronal growth and pruning^38^,^39^. The interplay of external disturbances and changes in normal brain growth encodes specific brain morphometry, making the onset age of Mn neurotoxicity an important factor to consider in interpreting the results. Prior imaging studies of gray matter maturation showed increased synaptic pruning and reduced gray matter density in adolescents’ brains^39^,^40^,^41^,^42^. Consequences of disturbances of this process are not well understood, albeit certain association of enlargement and neurobehavioral measures were reported by some studies. For example, based on the correlation results from 50 pre-adolescent children, Sandman *et al*. proposed an enlargement of the basal ganglia as a possible bio-marker of developmental impairment, with all three basal ganglia nuclei involved, and the putamen being preferentially affected^34^. In accordance with these findings, we detected significant enlargement in the basal ganglia, notably in the putamen. The enlargement of the caudate and putamen were also correlated with fine motor functions.

Alternatively, this enlargement pattern may be specific to Mn toxicity through drinking water. Researches on risk assessments of inhaled and ingested Mn reported quantitative differences of Mn tissue uptake via different dose routes^43^. In particular, inhaled Mn contaminated dusts that bypass normal gastrointestinal control system likely bind to transferrin as trivalent Mn, and thus are hard to eliminate by the liver and are easily accumulated in the brain via transferrin receptors^43^. Thus, the occupational Mn exposure investigated in previous studies may have a longer period of tissue uptake, and may eventually impact brain anatomy in a pattern different from other routes of Mn overexposure^35^,^36^. In our previous study on the same population, T1 intensity indices were also found to be different from those of adult studies^13^. These results, collectively, may indicate alternative Mn neurotoxicity mechanisms in children with waterborne Mn exposure. However, these preliminary hypotheses need further validation, including pharmacokinetic data on more subjects with different pathways of Mn overexposure.

The limitations of our study are as follows: First, we relied on Mn concentration in drinking water to define exposure groups. Mn water concentration was much more associated with hair Mn concentration (an exposure biomarker to Mn) than dietary Mn intake in a larger study from which our current subjects were selected^11^. However, it is possible that additional Mn exposure also came from air or food. Second, we did not explore the possible influence of other contaminants present in water. However, including iron concentrations in the regression model did not reduced the association between Mn concentration and the lower IQ scores, whereas other metals had very low concentrations (e.g. lead, arsenic). Third, our sample spans a range of ages from 9–15 years old, a period at which the brain is still rapidly developing, requiring the use of linear regression to control for the effect of age. Shaw *et al*. mapped the trajectory of BG development in 220 healthy subjects, and found that the growth of globus pallidus is close to linear^44^. In the same study, the trajectory of the striatum was found to be approximately linear from 7 to 14 years old, and the growth rate slows down afterward. In the current study, we have a total of 23 subjects, and only one of our subjects was over 14 years old at the time of scan. In order to avoid the over-fitting problem using nonlinear regression with a limited sample size, we adopted a simple linear regression to factor out the effect of age. This is recognized as one of the caveats of this study, and shall be optimized with better regression models in future larger datasets.

In the future, in addition to enrolling more subjects, we would also like to include more brain regions into our analysis. BG substructures investigated here are most likely the primary, but not the exclusive targets of Mn neurotoxicity. For example, alterations of the frontal cortex and corpus callosum have been reported in occupationally exposed adult welders^16^. These brain regions will be included in future.

## Methods

Brain T1-weighted MR images of 10 (mean age: 12.5, SD: 1.30 years) children with chronic exposure to Mn and 13 (mean age: 11.9, SD: 1.9 years) age matched controls were acquired with a 3T Philips Achieva X system. T1-weighted images were acquired using a 3D spoiled gradient-echo with inversion recovery preparation (repetition time (TR) = 8.1 ms, echo time (TE) = 3.7 ms, inversion time (TI) = 1005 ms, acquisition matrix = 240 × 240 × 160, resolution = 1 mm × 1 mm × 1 mm, SENSE factor = 1.5). An 8-channel phased-array head coil was used for signal reception, and head motion was minimized using cushions. Sequence duration was 7.5 minutes.

Selection criteria: For this present MRI investigation, carried out in 2010–2012, we selected children based on the concentration of Mn in the tap water of their residence, as measured in the initial study. We enrolled 13 children with low and 10 children with high Mn concentration in their drinking water (<30 and >100 *μg/L*, respectively). For each participant, an informed written consent was obtained from a parent, along with a written assent from the child. All MRI imaging was performed in accordance with guidelines and regulations of the Department of Radiology of the Centre Hospitalier de l’Université de Montréal (CHUM), using procedures that were approved by the Sainte-Justine Hospital, Notre-Dame Hospital and Université du Québec à Montréal research ethics committees. Written informed consent was obtained from the subject prior to the MR examination. The study was conducted in accordance with the Declaration of Helsinki. Mn concentration from the children in the low-exposure group ranged from 0.2 to 27 *μg/L* (median = 0.9, SD = 9) and those in the high-exposure group ranged from 103 to 264 *μg/L* (median = 145, SD = 54). Children from the two exposure groups were comparable in terms of age, sex, HOME scores, and full-scale IQ scores^13^, as shown in Table 2.

### Preprocessing

T1-weighted MR images for all the subjects are first bias corrected and skull stripped using the FSL software^45^. The preprocessed T1 data are linearly registered to one of the randomly chosen controls^46^. Three bilateral basal ganglia nuclei are manually traced on linearly aligned T1 images by a neuroradiology trainee using Insight Toolkit’s SNAP program^47^. The intra-rater percentage overlaps are 0.90 for the putamen, 0.91 for the globus pallidus, and 0.90 for the caudate. 3D surface representations of the 3 basal ganglia nuclei are constructed based on binary segmentations, and mesh grids are then built on the surfaces using our in-house conformal mapping program^32^. Subsequently, constrained harmonic based registrations are performed between an intermediate surface and each of the surface models, to obtain a one-to-one correspondence between vertices^32^. Then, volume based analysis and surface based analysis are performed, as discussed in detail in the following section.

### Volume based analysis

Among the various shape analysis methods on subcortical gray matter, the whole volume based analysis is the most intuitive one. Although volume based comparisons are generally limited in terms of detection and localization, they may provide preliminary insights into which structures may be affected. Here, we calculate the volumes of the 3 bilateral basal ganglia nuclei, to detect gross volume changes prior to the fine grained surface based morphometry analysis. A univariate *t-* test is performed on the volumes of each structure, and 10,000 permutations are employed to avoid the normal distribution assumption^48^,^49^. P- values for each of the tests are shown in Table 1.

### Surface based analysis

We used several shape measurements in our multivariate tensor based analysis. The first is medial axial distance (MAD), which represents the distance from a given surface vertex to the medial axis and represents the shape’s thickness at each point of its surface. The second is logged deformation tensor (*logS*). Specifically, the registration between each subject’s image and the template results in a displacement field 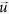. For each indexed vertex on subject’s surface, a Jacobian matrix 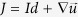 is computed, where *Id* is the identity matrix. In tensor-based morphometry, vertex-wise Jacobian matrices, or their derived functions are typically used as measurements in group analyses. In particular, the deformation tensor *S* (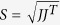), which can be expressed as a 2D ellipse at the center of each grid cell, captures directional difference at the corresponding location on the surface area as compared to a template^48^. The use of *S* largely preserves the information in shape changes, which is commonly lost when using the univariate determinant or trace of the Jacobian matrices (det *J* or *trJ*). To simplify computations, we use the logarithms of the deformation tensors (*logS*) instead of *S*, such that the projected elements form a vector space where standard Euclidean space formulae can be applied^50^.

According to our previous studies on populations with various age ranges^32^,^49^,^51^, the combination of MAD and logged deformation tensor (MADMTBM) has shown highest detection power compared to the widely used MAD or determinant based morphometry analyses (which detect surface area changes without the directional information). Thus, in this study, the surface based morphometry was mainly carried out through MADMTBM measures. For each vertex on the surface, the feature vector was defined as the combined logged deformation tensor and radial distance (4 × 1).

To compare the direction of alterations, i.e. whether there is an enlargement or a shrinkage in the high Mn exposed group compared with low exposed controls, we map the ratio of the mean *detJ* of the two groups at each vertex. The ratio maps are shown in Fig. 2. Furthermore, a multivariate analysis based on the 4 feature values (MAD and 3 values of the deformation tensor matrix) is performed to test whether the alteration is significant or not.

As previously stated, our subjects from the two exposure groups were comparable in terms of age, sex, home observation for measurement of the environment (HOME) scores, and full-scale IQ scores^13^. However, the effect of brain growth on BG morphometry from childhood into adolescence is not negligible^34^,^44^. Given the relatively large age range (from 9 to 15 years old) in our dataset, we use linear regression to factor out the effect of age. Linear regressions are carried out for each of the channel within the feature vector separately. Following this, Hotelling’s *T*^2^- tests are performed on the covaried feature vectors, as described in refs 33, 49 and 53.

Given the limited sample size of our study, we conduct vertex-wise permutation tests to avoid assuming a normal distribution. To do this, we shuffle the group labels and compare *T*^2^- values obtained as such with *T*^2^- values from the real data. For each vertex, 10,000 permutations are performed to assemble a null distribution of *T*^2^- values^53^. Vertex-wise corrected p-value maps for BG are shown in Fig. 3. In addition, each structure has 15,000 vertices on its surface, thus is inevitably subjected to multiple comparison errors. As a result, we also conducted 10,000 permutations over the whole structure surface to obtain a single *p*- value corrected for multiple comparisons. The overall p-values after multiple comparison correction are shown in Table 1. Both correction methods have been validated in previous multivariate tensor based morphometry studies with limited sample sizes^48^,^49^,^51^.

### Correlation Analysis

Pearson’s correlations are performed between mTBM measures of *detJ* and motor performance. Specifically, we use the Santa Ana Pegboard Test from the Neurobehavioral Core Test Battery to access hand dexterity and coordination^33^. Santa Ana scores were collected in our previous epidemiological study^11^, and have been shown to be most correlated to Mn exposure in the GP^13^. The vertex-wise correlation coefficients are shown in Fig. 4.

## Additional Information

**How to cite this article**: Lao, Y. *et al*. Mapping the basal ganglia alterations in children chronically exposed to manganese. *Sci. Rep.* **7**, 41804; doi: 10.1038/srep41804 (2017).

**Publisher's note:** Springer Nature remains neutral with regard to jurisdictional claims in published maps and institutional affiliations.

## Figures and Tables

**Table 1:** Group difference results of traditional univariate volume based analyses (VBA), and multivariate tensor based morphometry (mTBM) analyses on left (*l*-), right (*r*-), and combined (*c*-) basal ganglia structures: Putamen (Puta), Globus Pallidus (GP), Caudate (Cau).

Structures	Volumes (high exposure group)/*mm*^3^	Volumes (low exposure group)*/mm*^3^	*p* values VBA	*p* values mTBM
*l*-Puta	5075.8 ± 216.6	4725.2 ± 648.3	0.1460	0.0067^**^
*r*-Puta	4904.9 ± 311.5	4465.3 ± 1231.7	0.3686	0.1001^*^
*c*-Puta	9980.7 ± 447.5	9190.5 ± 1607.9	0.1801	0.0121^**^
*l*-GP	1298.2 ± 119.1	1221.2 ± 148.8	0.2338	0.0767^*^
*r*-GP	1213.5 ± 133.1	1152.6 ± 116.1	0.1878	0.2453
*c*-GP	2511.7 ± 235.5	2373.9 ± 258.2	0.2046	0.1045^*^
*l*-Cau	4156.4 ± 346.0	3921.4 ± 477.7	0.1759	0.1663
*r*-Cau	4431.9 ± 344.4	4234.9 ± 573.2	0.3124	0.1171
*c*-Cau	8588.2 ± 609.5	8156.3 ± 1037.0	0.2232	0.1396

All the *p*-values are corrected for multiple comparisons using structure-wide permutation testing with 10,000 permutations^48^,^49^. Significance is set at *p* < 0.05, and significant values are marked using**. Low *p*-values implying trends are marked using*.

**Table 2:** Demographics of our subjects.

Subjects	Number	Median water Mn concentration (*μg/L*)	Age at scan	Gender	IQ	Santa Ana scores
Mn low exposure group	13	0.9 ± 9	12.5 ± 1.3	5M8F	101.3 ± 9.1	36.9 ± 7.6
Mn high exposure group	10	145 ± 54	11.9 ± 1.9	4M6F	105.0 ± 10.8	30.2 ± 5.9
